# Video-Based Automatic Baby Motion Analysis for Early Neurological Disorder Diagnosis: State of the Art and Future Directions

**DOI:** 10.3390/s22030866

**Published:** 2022-01-24

**Authors:** Marco Leo, Giuseppe Massimo Bernava, Pierluigi Carcagnì, Cosimo Distante

**Affiliations:** 1 Institute of Applied Sciences and Intelligent Systems (ISASI), National Research Council of Italy, Via Monteroni Snc, 73100 Lecce, Italy; pierluigi.carcagni@cnr.it (P.C.); cosimo.distante@cnr.it (C.D.); 2 Institute for Chemical-Physical Processes (IPCF), National Research Council of Italy, Viale Ferdinando Stagno d’Alcontres 37, 98158 Messina, Italy; giuseppe.bernava@cnr.it

**Keywords:** baby motion analysis, early diagnosis, neurodevelopmental disorders, machine learning, deep learning

## Abstract

Neurodevelopmental disorders (NDD) are impairments of the growth and development of the brain and/or central nervous system. In the light of clinical findings on early diagnosis of NDD and prompted by recent advances in hardware and software technologies, several researchers tried to introduce automatic systems to analyse the baby’s movement, even in cribs. Traditional technologies for automatic baby motion analysis leverage contact sensors. Alternatively, remotely acquired video data (e.g., RGB or depth) can be used, with or without active/passive markers positioned on the body. Markerless approaches are easier to set up and maintain (without any human intervention) and they work well on non-collaborative users, making them the most suitable technologies for clinical applications involving children. On the other hand, they require complex computational strategies for extracting knowledge from data, and then, they strongly depend on advances in computer vision and machine learning, which are among the most expanding areas of research. As a consequence, also markerless video-based analysis of movements in children for NDD has been rapidly expanding but, to the best of our knowledge, there is not yet a survey paper providing a broad overview of how recent scientific developments impacted it. This paper tries to fill this gap and it lists specifically designed data acquisition tools and publicly available datasets as well. Besides, it gives a glimpse of the most promising techniques in computer vision, machine learning and pattern recognition which could be profitably exploited for children motion analysis in videos.

## 1. Introduction

Neurodevelopmental disorders (NDD) are impairments of the growth and development of the brain and/or central nervous system. They encompass several conditions, including intellectual developmental disorders, communication disorders, Autism Spectrum Disorder (ASD), Attention Deficit Hyperactivity Disorder (ADHD), specific learning disorders, and motor disorders. The prevalence has been increasing during the last two decades and it has been recently proven that preterm infants have an increased risk of neurodevelopmental disorders [1]. The human brain takes about forty years to reach its full-blown adult configuration, and then, assessments need to be age-specific, that is, the assessment techniques and assessment criteria should be adapted to the age-specific properties of the infant brain [2]. Anyway, there is a growing appreciation that the origins of these disorders are at the earliest stages of brain development, even prenatally [3]. The early origin of the neurodevelopmental disorders would potentially allow their early detection, and hence, an early onset of intervention, that is, intervention in a time window characterized by high neural plasticity. Gold standard methods exist in clinical practice for early diagnosis of NDD and they have been very well described in a recent survey paper [2]. Concerning motor assessments, three methods are commonly used to predict outcome: general movement assessment (GMA) [4], the Test of Infant Motor Performance (TIMP) [5] and the Infant Motor Profile (IMP) [6]. The GMA method provides an assessment of the spontaneous movements, called General movements (GMs), that are present from early fetal life onwards until the end of the first half a year of life. GMs are complex, occur frequently, and last long enough to be observed properly but, if the nervous system is impaired, GMs lose their complex and variable character and become monotonous and poor. Normally, they are the predominant movement patterns in an awake infant at 3 to 5 months [7]. IMP method evaluates motor abilities, movement variability, ability to select motor strategies, movement symmetry, and fluency. The IMP consists of 80 items and is applicable to children from 3 to 18 months. The TIMP method assesses the posture and selective control of movement needed by infants under four months of age for functional performance in daily life. Many studies demonstrated that cerebral palsy (CP) or autistic (ASD) children show disturbances of movement that could be detected clearly at the age of 4–6 months, and sometimes even at birth [8]. Attention Deficit Hyperactivity Disorder (ADHD) was found to be predicted in the first years of life by delays in gross motor milestones, abnormal GMs and less motor maturity [9,10,11]. Nearly half of the pediatric chronic pain patients suffer from comorbid mental health disorders, including mood and anxiety disorders, autism and ADHD. As a consequence, also monitoring pain in infants can help to predict NDD [12]. An overview of early behavioural markers for neurodevelopmental disorders in the first three years of life can be found in [13].

In the light of the aforementioned clinical findings and prompted by recent advances in hardware and software technologies, several researchers tried to introduce automatic systems (which should overcome differences in movements assessments of raters with various levels of experience [14]) to analyse the baby’s movements, even in cribs.

Most of the existing technologies for baby’s motion analysis are based on contact physical sensors [15]. They include force sensors, accelerometers, gyroscopes, extensometers, inclinometers, goniometers, electromyography [16]. Physical sensors assure high temporal resolution and very high accuracy but their use is discouraged by the sparsity of spatial data, difficulties to get consistent positioning and possible modifications of the behaviours to be observed. Alternatively, active/passive visual markers can be positioned on the children and acquired by optical devices. Their use is discouraged by the difficulties to get consistent positioning and then by long set-up times [17]. In fact, wearable physical sensors may alter baby movement productions. Under this perspective, markerless video-based approaches, which leverage data contents acquired from RGB cameras or Depth devices (e.g., Microsoft Kinect/Intel RealSense, etc.) in an ecological way (i.e., without any additional elements in the scene), become much more attractive for a reliable assessment of movements in children [18]. On the other hand, they require much more computational efforts and this brought researchers to initially concentrate on specific or easily detectable movements (i.e., exhibiting periodicity), for example, for spotting in real-time the occurrences of anomalies and providing prompt warnings to parents or healthcare staff [19]. Typical examples are systems that detect the presence or absence, respectively, of periodic movements of parts of the body—e.g., the limbs in case of clonic seizures and the chest/abdomen in case of apneas [20]. Other examples refer to tools for Neonatal Intensive Care Units (NICU) for detecting discomfort moments [21] and to estimate respiratory rate [22,23,24]. Most recently, methodological and technological progress in computer vision, machine learning and pattern recognition introduced disrupting improvements in automatic human activity recognition in videos (even relying on data acquired by handheld devices, such as smartphones [25]) and enabled the development of various automated applications in different fields, such as security and surveillance, healthcare, sports, home automation and recommender systems [26,27]. The baby’s motion analysis in videos for the early diagnosis of NDD benefited from this scientific fervor as well. Some of the related works dealing with GMA issues have been recently summarized in three systematic searches of papers [28,29,30] whereas those addressing the specific clinical task of detecting ASD features have been reviewed in [31]. The aforementioned survey papers are very interesting but they gave a task-specific (e.g., GMA or ASD assessment) view of the exploited computer vision and machine learning methods. This makes it difficult to understand how proposed approaches can be transferred to other tasks, involving different disorders and ages. A more global and structured vision of the problem would certainly be desirable to incentive research and its applicative effects but, unfortunately, to the best of our knowledge, the literature lacks a manuscript giving a broader overview of the video-based analysis of children motion for assessment of NDD. This paper fills this gap by providing a survey on advanced computational methods for early NDD diagnosis starting from temporal sequences of 2D/3D data. Besides, available software tools and public datasets are also considered. It gives also a glimpse of the most promising techniques in computer vision, machine learning and pattern recognition which could be exploited for children motion analysis in videos. The rest of the paper is organized as follows: Section 2 introduces a taxonomy to classify existing approaches in the scientific literature for movement assessment in children for early NDD diagnosis. Then, Section 3.2 introduces existing tools for data acquisition, collection and labelling whereas Section 4 discusses approaches for assessment of movements in newborns, infants and toddlers. The subsequent Section 5 provides a glimpse of the very latest methodologies for movement analysis which could be transferred in the considered domain of the early detection of NDD in children. Finally, Section 6 concludes the paper.

## 2. Taxonomy

Different categorisations of works dealing with the analysis of movements of infants for early NDD diagnosis can be carried out. The most straightforward one is based on the technology used to acquire input video data: RGB cameras, depth sensors or both. Acquisition devices can be handheld such as smartphones or fixed such as surveillance cameras. Another categorisation option relies on the acquisition conditions. Some works consider setups in which the children are in a hospital or an NICU. In these cases, children are in cribs, generally in the supine position, and then, acquisition devices and algorithms are designed to work properly according to this. Self-occlusions and other issues should be addressed to properly acquire the whole-body shape (for example, due to wearing diapers or sensors for monitoring vital parameters). Sometimes additional constraints are required to improve their robustness such as bedsheets of uniform colour and so on. Other works deal with children monitoring in more unconstrained environments such as homes and treatment centres. Acquisitions were carried out while adults are close to the child who can interact with the objects in the scene (e.g., puppets, tablets, etc.). Occlusions often happen and also lighting conditions change, sometimes even during the same acquisition session. Children can assume any possible posture (seating, standing, lying, etc.).

Alternative taxonomies can be pivoted either on the computer-vision/machine learning tasks actually addressed (data collection and labelling, static pose estimation, spatiotemporal modelling of motion, action recognition) or on the high-level clinical tasks pursued (ASD, CP or ADHD detection, pain quantification, monitoring rehabilitation/training sessions, etc.). All the aforementioned categorisations are certainly valid but, since the assessment techniques and criteria have to be adapted to the age-specific properties of the infant’s brain, in this paper, the categorisation will be carried out considering the age of involved children. It is the main element that pilots the architectural and algorithmic choices: acquisition setups depend on the acquired ability to walk; motion patterns are related to the age (according to previously mentioned assessment theories) and as consequence algorithms have to capture age-specific motion features. In the light of the above, in this paper, three different age ranges are considered to categorise related works in the literature: newborns (up to 2 months old), infants (2 months to 1-year-old) and toddlers (from 1 to 4 years old).

**Figure 1:**
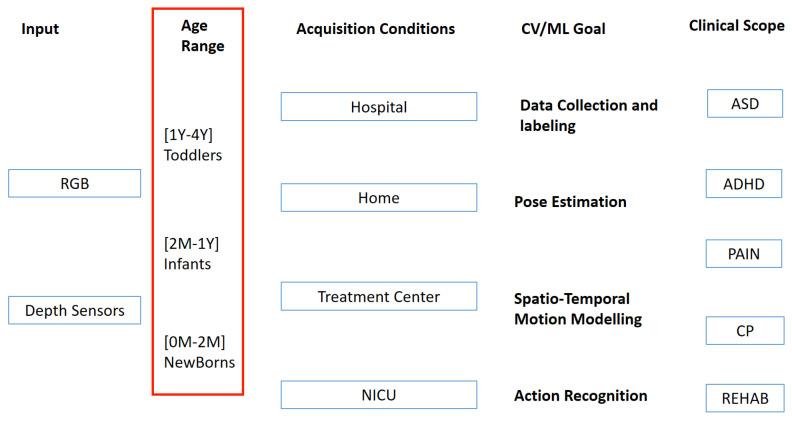
Introduced taxonomy.

Figure 1 summarizes the possible categorisation options (the chosen one is circled in red).

## 3. Data Acquisition, Collection and Labelling

The first steps towards the design of any video-based framework, for automatic motion analysis, are the setup of the acquisition devices, the secure collection and the privacy-preserving storage of data and, eventually, their annotation by experts in order to feed supervised machine learning algorithms. Under these premises, in the following subsections, existing acquisition tools and the most relevant annotated benchmark datasets specifically designed for baby motion analysis are described.

### 3.1. Acquisition/Recording Tools

It is quite difficult to find tools properly designed and set up for video-based analysis of children’s motor performance. In particular, two systems are described in the following. In chronological order, the first one is the AVIM system [32], a monitoring system developed in C# language using the OpenCV image processing library and specifically designed for an objective analysis of infants from 10 days to the 24th week of age. It acquires and records images and signals from a webcam and a microphone but also allows users to perform both audio and video editing. Very useful functionalities are the possibility of adding notes during the recording and to play/cut/copy and assess on-the-fly the sequences of interest. Besides, it extracts from the image the 2D position of the body segments to help the study of the movements according to amplitude, average speed and acceleration. The body analysis can concentrate either on the lower body, based on three points only, or on the full body by taking into account 8 points (right shoulder, left shoulder, left hand/wrist, base of the sternum, pubis/genitals, tight foot/ankle, left foot/ankle). It is worth noting that in both modalities (lower body or full body), all the points are manually placed and then tracked over time in order to extract some motion parameters according to the clinical literature are automatically extracted [33]. Some acoustic parameters (and related statistics) are automatically estimated as well (fundamental frequency, first two resonance frequencies of the vocal tract, kurtosis, skewness and time duration of each cry unit).

The second device deserving a mention is MOVIDEA [34] which has been designed for semi-automatic video-based analysis of infants’ motor performance. It includes a camera placed 50 cm above the child, at chest height, and software designed to extract kinematic features of limbs of a newborn (up to 24 weeks old) at home while lying on a bed, upon a green blanket. A Graphical User Interface completes the system and it allows the software operator to interact with the system. At first, the operator has to identify the limb by selecting the central point of the region of interest (i.e., hand, foot). The system then tracks the selected point frame by frame using the Kanade–Lucas–Tomasi algorithm [35] and movement features of extracted trajectories are compared with the reference ones for the identification of pathological motion patterns [36].

### 3.2. Publicly Available Datasets

In general, there is a lack of publicly available benchmark databases specifically built for children movement analysis. This is mainly due to privacy and security considerations that pose restrictions on ethics approval making this way very difficult to train robust models from scratch. Anyway, some exceptions exist. For the purpose of this survey, three different types of publicly available databases can be mentioned depending if they are oriented to build up models aiming at:

- Estimating the child pose;
- Comparing normative behaviours to those of monitored children in order to suggest further investigations;
- Recognizing atypical behaviours in order to directly get an NDD diagnosis.

Concerning pose estimation in newborns, the babyPose dataset [37] contains data relevant to preterm children’s movement acquired in NICUs. The data consist of 16 depth videos recorded during the actual clinical practice. Each video consists of 1000 frames (i.e., 100 s). The dataset was acquired at the NICU of the Salesi Hospital, Ancona (Italy). Each frame was annotated with the limb-joint location. Twelve joints were annotated, i.e., left and right shoulder, elbow, wrist, hip, knee and ankle.The database is freely accessible at http://doi.org/10.5281/zenodo.3891404 (accessed on 10 December 2021).

A benchmark dataset for a standardized evaluation of systems for pose estimation in infants has been made available for research purposes at http://s.fhg.de/mini-rgbd (accessed on 10 December 2021), namely the Moving INfants In RGB-D (MINI-RGBD dataset) [38] data set. It contains images of infants up to the age of 7 months lying in supine position, facing the camera. It has been created using the Skinned Multi-Infant Linear body model (SMIL) [39], a system able to build realistic infant body sequences (with both RGB and depth images) and to provide also a precise ground truth 2D and 3D joint positions. The dataset was bolstered by a recent study in [40] proving that movement assessment from videos of computed 3D infant body models is equally effective compared to the rating on conventional RGB videos. In particular, the MINI-RGBD dataset consists of 12 sequences of continuous motions (640×480 resolution at 25 FPS), each 1000 frames long. The sequences are divided into different levels of difficulty: lying on back, moving arms and legs, mostly besides the body, without crossing, (ii) medium: slight turning, limbs interact and are moved in front of the body, legs cross, and (iii) difficult: turning to sides, grabbing legs, touching the face, directing all limbs towards camera simultaneously.

A hybrid synthetic and real infant pose is the so-called SyRIP dataset [41]. The dataset includes a diverse set of real and synthetic infant images, which benefits from (1) appearance and poses of real infants in images from the web (from YouTube and Google Images), and (2) the augmented variations in viewpoints, poses, backgrounds, and appearances by synthesizing infant avatars. The dataset is available at https://github.com/ostadabbas/Infant-Pose-Estimation (accessed on 10 December 2021).

Concerning ‘normative’ reference datasets, the MIA dataset (https://vrai.dii.univpm.it/mia-dataset (accessed on 10 December 2021)) consists in the state vector (“in movement” or “not in movement" for each of the 4 limbs), along with the corresponding timestamp, derived from depth measurements collected by an RGB-D sensor placed perpendicularly above the child (a male hospitalized in an NICU) lying in a supine position on the crib, at a distance of 70 cm normally directed to the subject. Unfortunately, no video was provided for privacy reasons but, anyway, the provided state vector could be used for training models to be subsequently tested on data extracted from videos.

Recently, a ‘normative’ reference database of infant movements has been created using 85 videos found online [42]. Two physical therapists estimated the age of the infants. Estimated mean age was 9.67 weeks and standard deviation 6.26 weeks. Using this normative database, OpenPose tool [43] and a Gaussian estimator, the authors calculated how much 19 high-risk children deviate from the typical movements of healthy infants. Code and data referenced in the manuscript are provided at https://github.com/cchamber/Infant_movement_assessment/ (accessed on 10 December 2021).

The most used publicly available dataset of videos for Autism Diagnosis is named Self-Stimulatory Behaviours (SSBD dataset) [44]. It consists of videos of children exhibiting self-stimulatory (‘stimming’) behaviours commonly used in autism diagnosis. These videos, posted by parents/caregivers on public domain websites, are collected and annotated for the stimming behaviours. These videos are extremely challenging for automatic behaviour analysis as they are recorded in uncontrolled natural settings. The dataset contains 75 videos with an average duration of 90 s per video, grouped under three categories of stimming behaviours: arm flapping, head banging, and spinning. The dataset, the terms of use and the Matlab script file to generate baseline results (making use of a combination of STIP, HIG/HOF and Bag-of-Words, plus SVM for classification) are available at https://rolandgoecke.net/research/datasets/ssbd/ (accessed on 10 December 2021).

The Multimodal Dyadic Behaviour (MMDB) dataset [45] is a unique collection of multimodal (video, audio, and physiological) recordings of the social and communicative behaviour of toddlers (aged 15–30 months). The dataset contains 160 sessions of 5-minute interaction from 121 children. All multimodal signals are synchronized, including 2 frontal view Basler cameras (1920 × 1080 at 60 FPS), an overhead view Kinect (RGB-D) camera, 8 side view and 3 overhead view AXIS cameras (640 × 480 at 30 FPS), an omnidirectional and a cardioid microphone, 2 wireless lapel microphones, 4 Affectiva Q-sensors for electrodermal activity and accelerometry, worn by both the adult and the child.

Another dataset commonly used to train and test machine learning algorithms to classify ASD-related behaviours is the one introduced by Tariq et al. [46]. This dataset was collected through a mobile web portal built up by some researchers at Stanford University and it contains 116 short home videos of children (age range 2–4 years old) with autism and 46 videos of typically developing children.The de-identified data have been made available at the following GitHub repository and include the primary dataset and the validation dataset: https://github.com/qandeelt/video_phenotyping_autism_plos/tree/master/datasets (accessed on 10 December 2021).

Recently, the DREAM Dataset [47] has been also made publicly available at https://snd.gu.se/sv/catalogue/study/snd1156/1/1# (accessed on 10 December 2021). It consists of behavioural data recorded from 61 toddlers diagnosed with autism spectrum disorder collected during a large-scale evaluation of Robot Enhanced Therapy (RET). The public release of the dataset comprises body motion, head position and orientation, and eye gaze variables, all specified as 3D data in a joint frame of reference. In addition, metadata including participant age, gender, and autism diagnosis (ADOS) variables are included.

Finally, depth videos templates of autistic repetitive behaviours (e.g., hands on the face, hands back, tapping ears, hands stimming, hand moving front of the face, toe walking, walking in circles, etc.) were collected in the dataset 3D-Autism Dataset (3d-AD) [48]. Each action has been repeated at least 10 times with non-autistic people. The depth maps have been captured at a rate of 33 frames per second with a Kinect-v2 camera.

Table 1 shows the main properties of the aforementioned publicly available datasets.

## 4. Methods and Systems for Movement Assessment

**Figure 2:**
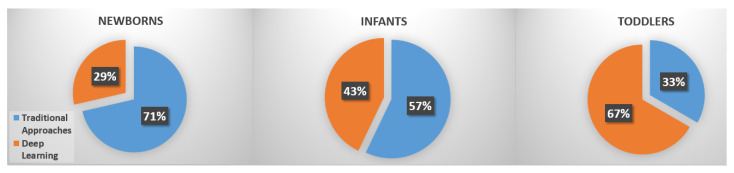
Percentages of published papers with respect to Age Range taxonomy.

Searching for the considered topic, 20 works were found in the literature, quite equally distributed in the three age ranges (7 for newborns, 7 for infants and 6 for toddlers). It is worth noting that most of the works dealing with newborns leverage traditional computer vision methods (5 of 7). Concerning movements analysis in infants, this trend persists but only 3 of 7 works did not use deep learning-based approaches. Finally, when involving toddlers, the majority of the works made use of deep learning strategies (only 2 of 6 did not). In Figure 2, a pie chart representing the impact of deep learning in each age range is reported. It is possible to observe that when dealing with newborns, most of the solutions made use of ’traditional approaches’, i.e., based on handcrafted features and/or shallow classifiers. The percentage of deep-learning-based solutions increased when infants were involved and became predominant while handling toddlers. This is not surprising since most of the approaches made use of pre-trained models (on adults), and then, it is straightforward to use them on walking children instead of on children in supine pose (in a bed).

**Figure 3:**
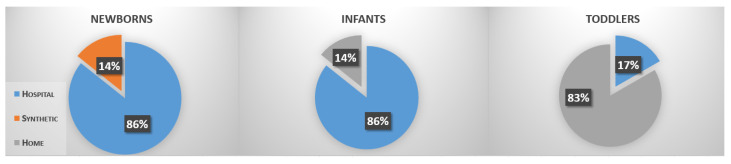
Percentages of published papers with respect to setup taxonomy (home, hospital, etc.).

Another preliminary consideration could be made about acquisition settings. In Figure 3, the percentages of published papers with respect to setup taxonomy (home, hospital, etc.) is reported. Methods dealing with newborns were mainly applied in hospital/NICU settings with only one work handling just synthetic data. Home settings were partially used for infants and in most of the experiments on toddlers. Once again, this is not surprising since as children grow, it becomes necessary to assess them in environments where their behaviour is not conditioned by the context.

The following subsections will detail the related works found in the literature.

### 4.1. Newborns

Newborn usually refers to a baby with an age from pre-term to approximately 9 weeks post-term. According to [49], that is the so-called ‘writhing’ age. Movements in this writhing age can be categorised as Writhing Movements (WM), Poor Repertoire (PR), Cramped Synchronised (CS) or Chaotic Movements (CM). These categorisations represent various levels of quality to the typical movements during this age period. The presence of persistently cramped synchronized writhing movements, followed by the absence of fidgety movements, is the strongest predictor for poor neurodevelopmental outcomes [4]. In particular, it has been reported that observation of poor repertoire pattern seems to be associated with minor neurological dysfunctions [50] and cramped-synchronized movements is highly predictive of the development of cerebral palsy [51]. Current studies indicate that the GMA is the most sensitive and specific test available to allow early detection of CP [52]. These kinds of assessments are carried out especially in the *NICUs*, and they need to accurately identify those infants most at risk. Early video-based works in this research area used fast recognition of key points to find the 3D positions of body joints in single depth images [53,54]. The tuning of model parameters exploited poses that are not typical for infants and their specific motions, but a series of synthetically generated baby-like poses was also added in order to reduce that bias. Tests were carried out on babies at the age of 3 months who were always filmed from above so that the main body axis is displayed vertically in the camera image.

In [55], a deformable part-based model was exploited to detect the body parts (by skeletonization) of children aged from 2 weeks to 6 months. Then, angles for joints are computed and tracked temporally in a video sequence to describe movements. Although the authors evaluated the accuracy in the estimation of joint positions and movements encoding without a specific clinical application, they asserted that it was specifically designed to evaluate the patient’s poses and movement during therapeutic procedures (e.g., Vojta techniques [56]) aimed at early diagnosis of cerebral palsy, spinal scoliosis, peripheral paralysis of arms/legs, hip joint dysplasia and various myopathies.

Authors in [57] introduced a system relying on a multimodal recording setup consisting of two HD cameras, two Kinect and sensors for pressure measurement. Attributes used in audio/video analysis, such as optical flow, zero-crossings rate, harmonics-to-noise ratio and jitter are computed for children in their first 4 months of life and compared with those of children (of the same age) having a typical development and with diagnosed conditions of interest. To this end, logistic regression from multidimensional data was exploited.

The study in [58] attempted to automatically detect writhing movements instead. The study involved newborns on the second and third days of life. Different feature extraction strategies and traditional machine learning algorithms were exploited for writhing movement detection. Based on automatically detected writhing movement percentages in the videos, infants are classified as having a good level of writhing movements or as having a poor repertoire, i.e., a lower quality of movement in relation to the norm.

Even if the use of deep learning techniques in this application context is not easy, due to the lack of annotated data, recently, some solutions based on convolutional neural networks (CNN) have nevertheless been proposed. An approach to preterm infants’ limb pose estimation that features spatiotemporal information to detect and track limb joints from depth videos with high reliability was proposed in [59]. The depth camera (model Astra Mini S-Orbbec (https://orbbec3d.com/astra-mini-series/ (accessed on 10 December 2021)), with a frame rate of 30 frames per second and image size of 640×480 pixels) is positioned at 40 cm over the infant’s crib in order to not hinder health-operator movements.Limb-pose estimation is performed using a deep-learning framework consisting of detection and regression CNNs for rough and precise joint localization, respectively. The CNNs are implemented to encode connectivity in the temporal direction through 3D convolution. Assessment of the framework was performed through a comprehensive study with sixteen depth videos acquired in the actual clinical practice from sixteen preterm infants. The proposed solutions can be exploited for diagnostic support, e.g., to classify abnormal limb movements.

**Figure 4:**
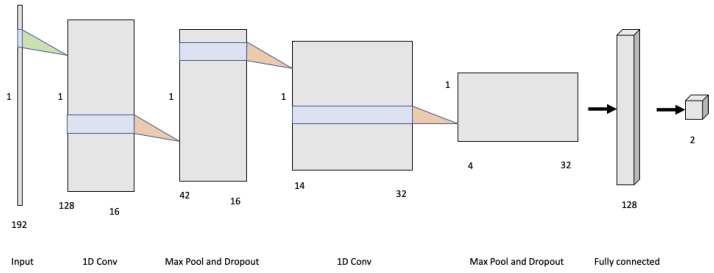
1D convolutional neural network architecture exploited in [60] for labelling observed movements as indicative of typically developing infants (Normal) or that may be of concern to clinicians (Abnormal).

Finally, authors in [60] proposed five deep-learning-based frameworks to classify infant body movement based upon the pose-based features which consisted of histogram representations describing different aspects of the extracted pose-based features. The final aim was to automatically label observed movements as indicative of typically developing infants (Normal) or that may be of concern to clinicians (Abnormal). Synthetic data from the MINI-RGBD dataset were used and an accuracy of over 90% was achieved by using histograms of joint displacement and orientation as features and 1D convolutional neural network architectures exploited as reported in Figure 4.

Table 2 Summarizes works dealing with movements assessment in newborns.

### 4.2. Infants

After 9 weeks of age, in the proper development of the infant, writhing movements are replaced by fidgety movements that are present continuously in an awake infant. They involve the whole body and are circular movements of small amplitude and variable acceleration and they disappear after 15–20 weeks, along with the appearance of voluntary movements. As a consequence, four types of movements can then appear at this age: writhing movements (WMs), fidgety movements (FMs), poor repertoire (PR) and cramped-synchronized (CS). Studies using GMA suggest that the absence of fidgety movements between 9 and 15 weeks is the best criterion for early identification of CP and other developmental disorders [61].

To this aim, the system proposed in [62] calculated magnitude, balance and rhythm of movements by video analysis. Movements are detected by background subtractions and frame differences, whereas their classification is performed, based on the clinical definition, by using a feedforward-type neural network that includes Gaussian mixture models in a log-linearised form, enabling the estimation of the probabilistic distribution of a given sample dataset. This way, the system automatically classified the input motion images of infants during GMs into one of the four above mentioned types (WM, FM, PR or CS). Nineteen infants, including some with LBW (Low Birth Weight), were recorded either at home or hospital while standing in a crib. The movement of infants is measured using a video camera fixed directly above and parallel to the crib surface, which is covered by a unicolour fabric spread.

Automated detection and classification of presence vs. absence of FMs was the aim in [63] instead. A dataset of 2800 five-second snippets was annotated by two well-trained and experienced GMA assessors. Using OpenPose, the infant’s full pose was recovered from the video stream in the form of a 25-points skeleton. This skeleton was used as an input vector for a shallow multilayer neural network in order to discriminate fidgety from non-fidgety movements.

The abovementioned works aimed at classifying movement types. However, higher-level reasoning can be introduced to directly scoring the risk of a disorder in observed infants. For example, in [64], a machine-learning model for early Cerebral Palsy (CP) prediction based on infant video recordings in NICUs has been proposed. The model was designed to assess in videos, acquired by a commercially available digital video camera, the proportion (%) of CP risk-related movements. The first processing step in the model consists of a time-frequency decomposition, by multivariate empirical mode decomposition (MEMD) and Hilbert–Huang transformation [65], of the movement parameters extracted for six body parts (arms, legs, head, and torso). Subsequently, each 5-second period in the video was clustered into 5 composite scores which were used in a linear discriminative analysis to classify movements typically found in children with or without CP. The model was developed and tested on video recordings from a cohort of 377 high-risk infants at 9–15 weeks corrected age to predict CP status and motor function (ambulatory vs. non-ambulatory).

**Figure 5:**
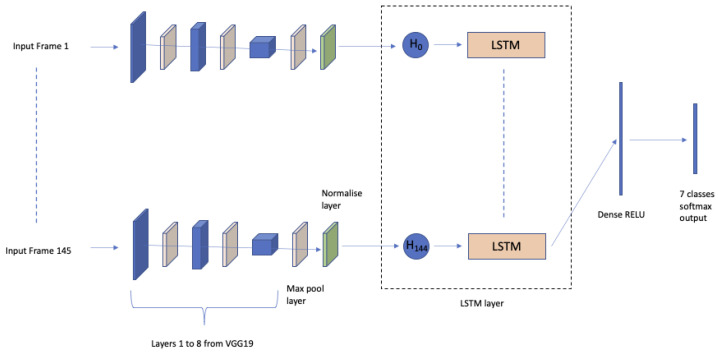
Normal or abnormal movement classification by means of VGG for feature extraction and LSTM for temporal modelling as proposed in [66].

Instead of using handcrafted features and a decision tree, in [66], image features were taken from Layer 8 of VGG19 [67], passed through a max-pooling layer and normalized before being input to a Long short-term memory (LSTM) [68] layer for classification of the image sequence. The classification outcomes label each sequence as containing normal or abnormal movements. The model, constructed using a transfer learning approach, is represented in Figure 5. Experimentally, on a dataset of videos taken from 80 CP and 135 normal subjects, it has been proved that it can classify normal videos with great confidence but struggles with intermittent classes. Classification of these borderline classes is also a difficult task for experts. How much video is required before an LSTM can identify the presence of CP is a key research question. It is hypothesized that, as a minimum, the videos should be no shorter than required for an ‘expert’ to make a positive identification.

Infants evaluated as at high-risk of CP, with an age of 12–24 weeks post-term, were examined in [69]. Parents and families were asked to video-record their baby through the In-Motion-App by a smartphone. Infants in videos were assessed by a motion tracker algorithm that consists of a convolutional neural network trained on 7-body points on about 15K video frames on high-risk infants. The final goal was to predict CP. This has been the first automatic infant body point tracker tested on video recordings from hand-held smartphones.

Some recent work addressed also the problem of making the proposed frameworks fully interpretable, i.e., providing an automatically generated visualization capable of relaying pertinent information to the assessor. To this aim, the framework in [70] takes video as the input and analyses the movement of individual body parts to determine if FMs are present or absent, subsequently identifying normal or abnormal general movements from segments of the sequence. The 2D skeletal pose is detected on a per-frame basis using OpenPose, hence, each pose is divided into different body parts and each body-part sequence is processed by a specific branch to learn a part-specific spatiotemporal representation (using LSTM). Finally, the outputs from all the individual body-part streams are concatenated and fed to the classifier. The label predicted by the classifier is then returned to the user as text message printed on the original video by a specific visualization module.

Infants tend to shake their heads, extend their arms/legs, and splay their fingers when they experience pain. Therefore, body movement is considered the main indicator in several pediatric scales [71].

For example, in [72], infants having an average gestational age of 36 weeks were recorded before, during and after an acute episodic painful procedure. It was then proved that the amount of body motion presents a good indication of the infant’s emotional state. The amount of motion in each video frame was computed by summing up the motion’s image pixels and it was used as the main feature for classification by a threshold (pain-related movement or no pain-related movement).

Table 3 Summarizes works dealing with movements assessment in infants.

### 4.3. Toddlers

The term toddlers refers to children who have recently learnt to walk, i.e., having 1–2 years of age. Differently from newborns and infants, when toddlers are involved, acquisition conditions are more challenging since generally the child is acquired while freely moving and adults (and even other agents such as robots) are present, with the child being one of the smallest actors in the scene. On the other side, distinctive features of NDD can be more evident and a computer-based diagnosis becomes more feasible and reliable. In other words, for toddlers, the computer-aided clinical goal is to distinguish between children with and without NDD. Reliability and feasibility depend on the acquisition environment that could be domestic or some rehabilitative centre.

The problem of analyzing actions performed in a pediatric rehabilitation environment has been addressed in [73]. Automatically recognizing actions can help to assess infants’ mobility skills and to understand if and how adults and socially assistive robots can promote their mobility. The paper proposes a multiview action classification system based on Faster R-CNN and LSTM networks that fuses information from different views by using learnable fusion coefficients derived from detection confidence scores. Pretrained deep features and detection annotations for training were used. The system is view-independent, learns features that are close to view-invariant, and can handle new or missing views at test time. The approach was tested on a small dataset (2 subjects, 10–24 months old) and on an extended dataset (6 subjects, 8–24 months old) for four action classes (crawl, sit, stand and walk).

Some interesting works concentrated on the possibility to disambiguate typically developing vs. autistic subjects using an ML approach operating on video sequences of simple executing gestures.

In [74], a Random Forest classifier [75] was trained to distinguish between Typical/Atypical Development and Autism Spectrum Disorder/Speech and Language Conditions. An interesting part of the study determined the impact of each video’s annotations on the classifier’s predicted label for that video by a unified approach inherited by the game theory.

Videos of a child’s daily activities at home were analysed in [76]. Common categories of daily activities include “play alone”, “play with others”, “mealtime”, and “parents’ concerns”. At first, the authors re-trained a 2D Mask R-CNN network to make it more robust in identifying children pose. A nonlinear state estimation technique was then exploited to predict the locations of missing key points. Finally, behavioural features were extracted from key points trajectories over a short time frame and they were exploited to feed a binary classifier to distinguish between atypical vs. typical characteristics.

In [77], a computer vision classifier for detecting head banging in home videos has been proposed. The solution uses the well-known scheme (see Figure 5) with a time-distributed convolutional neural network (CNN) in which a single CNN extracts features from each frame in the input sequence, and these extracted features are fed as input to a long short-term memory (LSTM) network. The solution achieved a 90.77% F1-score on video clips from the SSBD dataset.

**Figure 6:**
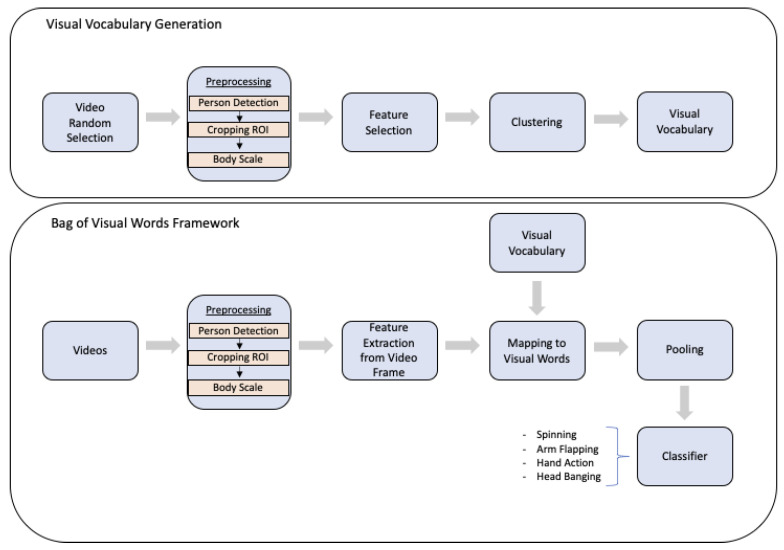
The innovative tool proposed in [78]. It follows a Bag-of-Visual-Words configuration for recognising 4 repetitive actions that are a potential indication of ASD disorder.

The aforementioned scheme (CNN+LSTM) has been exploited in [78] for building a baseline in recognising 4 repetitive actions (spinning, arms flapping, hand action, and head banging actions) that are a potential indication of ASD disorder. Besides, the authors introduced an innovative tool which follows a Bag-of-Visual-Words configuration as reported in Figure 6. It firstly performs a person detection and tracking module by the YOLOv3 detector, feature extraction by Histogram of Optical Flow (HOF), and data clustering by K-means. Then, each video input is coded in visual words that are finally classified by MLP. Inputs are videos of 3-years old children and they are captured by parents in daily living settings. The best algorithmic pipeline (among those tested in the paper) achieved 78% of accuracy. Anyway, the proposed pipeline had several drawbacks and it did not provide satisfying outcomes in natural settings. A reason is that only 2D pose/appearance descriptors and weak models for spatiotemporal information were involved. Besides, the number of videos for tests was limited (i.e., 141) and, in most of them, there was the issue of shaking camera held by the parents during the recordings. Finally, authors in [79] applied machine learning methods (Logistic Regression with varying regularization penalties, SVM Classifiers, attempting a wide range of kernels and hyperparameters and decision trees) on ratings of different indicative features of autism from home videos.

Table 4 summarizes works dealing with movements assessment in toddlers.

## 5. Recent Advances in Human Motion Analysis

As described in the previous sections, the approaches for assessing children’s movements rely on well-consolidated machine learning techniques. Some of them still rely on classical strategies (hand-crafted features and shallow neural networks) whereas the most recent and performing ones exploit deep learning for feature extraction or for providing end-to-end solutions. Anyway, research in machine learning is running ahead very fast [80], and thus, it could be of interest here to have a glimpse on the very latest methodologies for movement analysis which could be transferred in the considered domain of the early detection of NDD in children.

There are several possible research directions that could be pursued to improve existing frameworks aiming at computer-based early diagnosis of NDD by analysing video data. Among all, the tasks that have been attracting great attention from the machine learning and computer vision community are:

- Motion feature extraction;
- Human pose estimation;
- Extraction significant motion segments/temporal action localization;
- Human image completion;
- Action recognition and action quality assessment;
- Humans-objects interaction prediction/understanding;
- Spatiotemporal video representation;
- Interpretablilty of involved AI models.

Improving motion feature extraction could be the first pathway to explore for improving computer-aided diagnosis. To this aim, a rich and robust motion representation based on spatiotemporal self-similarity has been recently proposed [81]. Given a sequence of frames, the method represents each local region with similarities to its neighbours in space and time, enabling this way the learner to better recognize structural patterns in space and time. Code is available at https://github.com/arunos728/SELFY (accessed on 10 December 2021). Alternatively, the trainable neural module (MotionSqueeze) for effective motion feature extraction proposed in [82] could be exploited. Inserted in the middle of any neural network, it learns to establish correspondences across frames and convert them into motion features, which are readily fed to the next downstream layer for better prediction. Code is available at https://github.com/arunos728/MotionSqueeze (accessed on 10 December 2021).

In addition, improving human pose estimation could help the technologies for NDD diagnosis in children. To this aim, it is undoubtedly important to improve the localization quality of the regressed key point positions and this could be achieved by a multi-branch structure for separate regression (i.e., each branch learns a representation with dedicated adaptive convolutions and regresses one key point) as proposed in [83]. The code and models are available at https://github.com/HRNet/DEKR (accessed on 10 December 2021). An effective regression-based human-pose recognition method could be also carried out by building cascade transformers as suggested in [84] whose code has been made available at https://github.com/mlpc-ucsd/PRTR (accessed on 10 December 2021). Pose estimators still suffer from severe performance drop on corrupted images, and thus, some authors proposed to overcome this drawback by an adversarial data augmentation method together with a knowledge distillation module applied to transfer clean pose structure knowledge to the target pose estimator [85]. Code available at https://github.com/AIprogrammer/AdvMix (accessed on 10 December 2021). Operating on either pixel-level or key point-level transitions is good for analysing local and short-term motions of human bodies but not to handling higher-level spatial and longer-lasting temporal structures well.

Since children’s body is often partially occluded, useful approaches could be those solving human image completion, which tries to recover the human body part with a reasonable human shape from the corrupted region. In [86], a framework for recovering the human body parts by a reasonable topological structure of the human body has been introduced. The paper proposes a structure and texture memory bank to introduce more additional priors as compensation for the corrupted region.

Skeleton-based action recognition is a very common solution also in children movement analysis. A substantial improvement of this strategy has been recently proposed in [87] where a temporal-then-spatial recalibration method, named memory attention networks (MANs), has been deployed using a temporal attention-recalibration module and a spatiotemporal convolution module.

In recent years, a number of end-to-end approaches based on 2D or 3D convolutional neural networks (CNN) have emerged for video action recognition, achieving state-of-the-art results on several large-scale benchmark datasets. An in-depth comparative analysis of available approaches on video data framing adults is available in [88]. How they can impact the movement analysis of children and the recognition of their actions is less debated instead [89]. Furthermore, their impact in the specific context of early diagnosis of NDD is totally missing. In the following, some examples of end-to-end (E2E for short) deep learning-based methods that can potentially impact the NDD diagnosis are reported. An approach with a novel temporal-spatial pooling block for action classification, which can learn pool discriminative frames and pixels in a certain clip, has been recently proposed in [90]. Similarly, in [91], an efficient spatiotemporal human action recognition framework for long and overlapping action classes has been proposed. Fine-tuned pre-trained CNN models were exploited to learn the spatial relationship at the frame level whereas an optimized Deep Autoencoder [92] was used to squeeze high-dimensional deep features. A Recurrent Neural Network (RNN) with LSTM [93] was used to learn the long-term temporal relationships.

However, RNN suffers from non-parallelism and gradient vanishing; hence, it is hard to be optimized, and then, encoder-decoder frameworks based on transformers [94] are becoming popular. For example, in the solution introduced in [95], the encoder attached with a task token aims to capture the relationships and global interactions between historical observations. The decoder extracts auxiliary information by aggregating anticipated future clip representations. Therefore, a transformer can recognize current actions by encoding historical information and predicting future context simultaneously. The code is available at https://github.com/wangxiang1230/OadTR (accessed on 10 December 2021). Following this research trend, a Video Transformer (VidTr) model with separable attention for video classification has been proposed [96]. Compared with commonly used 3D networks, these frameworks are able to aggregate spatiotemporal information via stacked attention and provide better performance with higher efficiency [97]. Multiscale Vision Transformers (MViT) for video and image recognition could be another important architecture to test for children movement analysis. It connects the seminal idea of multiscale feature hierarchies with transformer models [98]. Code is available at https://github.com/facebookresearch/SlowFast (accessed on 10 December 2021). A pure-transformer-based model for video classification, drawing upon the recent success of such models in image classification has been proposed in [99]. The model extracts spatiotemporal tokens from the input video, which are then encoded by a series of transformer layers. Code was released at https://github.com/google-research/scenic/tree/main/scenic/projects/vivit (accessed on 10 December 2021).

Training temporal action detection in videos requires large amounts of labelled data, yet such annotation is expensive to collect. This could be more and more challenging in the case of children, and even more if NDDs have to be observed. Incorporating unlabelled or weakly-labelled data to train action detection models could help reduce annotation costs. In [100], authors designed an unsupervised foreground attention module utilizing the conditional independence between foreground and background motion and put it in a Semi-supervised Action Detection (SSAD) task.

In a real-world scenario, human actions are typically out of the distribution from training data, which requires a model to both recognize the known actions and reject the unknown. Different from image data, video actions are more challenging to be recognized in an open-set setting due to the uncertain temporal dynamics and static bias of human actions. This is even more true in the case of children and in particular when we want to identify subtle behavioural differences. To overcome this issue, some researchers [101] formulated the action recognition problem from the evidential deep learning perspective and proposed a novel model calibration method to regularize the training and to mitigate the static bias of video representation through contrastive learning [102]. Code and pre-trained models used in [101] are available at https://www.rit.edu/actionlab/dear (accessed on 10 December 2021).

Another task that could be of interest in analyzing children’s movement is related to the automatic Action Quality Assessment, i.e., analysing/quantifying how well an action (either spontaneous or voluntary) was performed. Assessing action quality is challenging since it has to rely on just subtle differences while performing. Mapping these differences, when found, in reliable scores is a difficult task as well. Regression strategies are commonly used to tackle this problem but they suppose to be able to extract a reliable quality score from a single video, ignoring the ineluctable large inter-video variations even when the action is performed by the same person. The consideration that relations among videos can provide important clues for more accurate action quality assessment during both training and inference inspired the work in [103]. The authors reformulated the problem of action quality assessment: instead of learning unreferenced scores, they aimed at regressing relative scores with reference to another video that has shared attributes (e.g., category and difficulty). Small intervals are considered in order to build a coarse-to-fine approach. In other words, they proposed a differential approach followed by a grouping strategy in order to achieve effective action scoring.

The PyTorch implementation of the method is available at https://github.com/yuxumin/CoRe (accessed on 10 December 2021).

Unfortunately, some children actions have a similar appearance and they require complex temporal-level relation understanding to be well analysed. Hence, a way to overcome this drawback could be to get an effective spatiotemporal video representation that could help to disambiguate them. Representing video structure as a space-time graph and discovering the discriminative sub-graphs is the solution proposed in [104]. This also leads to the elegant views of how to perform end-to-end learning of the discriminative sub-graphs, and how to nicely present the complexity of different actions in the reasoning process, which are problems not yet fully understood. The recent paper [105] studies the problem of learning self-supervised representations on videos. It presents a contrast-and-order representation framework for learning self-supervised video representation that can automatically capture both the appearance information within each frame and temporal information across different frames. Self-supervised video representation learning methods have been also addressed in [106] by two tasks to learn the appearance and speed consistency, respectively. Nevertheless, temporal modelling still remains challenging for action recognition in videos. To mitigate this issue, new video architectures with a focus on capturing multi-scale temporal information for efficient action recognition are being proposed. For example, in [107], an efficient temporal module that leverages a two-level difference modelling paradigm, assessing local and global motion, respectively, on short-term and long-term motion modelling, has been recently introduced. Code at https://github.com/MCG-NJU/TDN (accessed on 10 December 2021).

A list of the works mentioned in this section is shown in Table 5: the leftmost column indicates the referring works, the central one points out which tasks, among those involved in frameworks for the early diagnosis of NDD, have been improved. Finally, the methodological contributions that brought to the knowledge advancement are highlighted in the rightmost column.

One of the main issues when using machine learning approaches, especially in the medical domain, is to understand the rationale behind the prediction. This general problem is referred to in the literature as interpretable AI. Models are often seen as ‘black boxes’ in which the underlying structures can be difficult to understand. There is an increasing requirement for the mechanisms behind why systems are making decisions to be transparent, understandable and explainable. In medical scenarios, the cost of a simple prediction error could be significantly high, and thus, the reliance on the trained model and its capability to deliver both efficient and robust data processing must be guaranteed. Therefore, understanding the behaviours of machine learning models, gaining insights into their working mechanisms, and further generating explainable deep learning models have become essential and fundamental problems. Hence, another research line could be explaining the AI, and, in particular, making clearer how deep learning works for movement analysis when children are involved. To this aim, including visualization and uncertainty estimation can improve acceleration, robustness and stability making automatic tools actually exploitable in the clinical practice for early diagnosis of NDD. To this end, a starting reading could be the recent survey paper on explainable AI [115].

Another relevant issue to be furtherly investigated in order to improve computer-based diagnosis of NDD in children is the Motion Segment Extraction, which aims at detecting the temporal location of significant motion in the scene. To this aim, the authors in [108] incorporated higher-level reasoning of motion primitives by introducing a hierarchical motion understanding framework. They demonstrated also how to detect and extract significant motion segments that can be a crucial point in many diagnosis tasks. Code is available at https://sumith1896.github.io/motion2prog (accessed on 10 December 2021). A very strictly related issue is the temporal action localization, which is an important yet challenging task in video understanding. Typically, such a task aims at inferring both the action category and localization of the start and end frame for each action instance in a long, untrimmed video. They can rely either on pre-defined anchors, generated by different levels of supervision [110], or on anchor-free end-to-end trainable basic predictor [109]. Anchor-based methods generally provide a large number of outputs and require a heavy tuning of locations and sizes corresponding to different anchors. Instead, recently introduced anchor-free methods are lighter and get rid of redundant hyper-parameters. The code of the anchor-free approach proposed in [109] is available at https://github.com/TencentYoutuResearch/ActionDetection-AFSD (accessed on 10 December 2021).

Temporal action localization has been also addressed by an Expectation-Maximization (EM) framework that comprises Hidden Markov Models, MLP and self-supervised learning for action-level temporal feature embedding [116]. This way, it relaxes assumptions about the lengths of latent actions. Alternatively, in [111], an Action Unit Memory Network for weakly supervised temporal action localization was proposed. Two attention modules were designed to adaptively update the memory bank and to learn action units. Similarly, in [112], an attention based Multi-Label Action Dependency layer was introduced to improve action localization performance. The layer consists of two branches: a Co-occurrence Dependency Branch and a Temporal Dependency Branch to model co-occurrence action dependencies and temporal action dependencies. Code is available at https://github.com/ptirupat/MLAD (accessed on 10 December 2021). Finally, the problem of human–object interaction detection is very important when observing children’s behaviours (especially for toddlers). It has been effectively addressed by a unified model (exploiting a transformer unit and a cascade detection over multi-scale feature maps) to jointly discover the target objects and predict the corresponding interactions in [113] and by Asynchronous-Sparse Interaction Graph Networks in [114]. Code available at https://github.com/scwangdyd/ (accessed on 10 December 2021) and https://github.com/RomeroBarata/human_object_interaction (accessed on 10 December 2021) respectively.

## 6. Conclusions

This paper summarizes the most relevant works on movement analysis in young children (aged 0–3) employing mainly machine learning techniques and starting from image/video data. The work was motivated by the observation that existing review papers dealt with technologies based on physical sensors. Actually, a few works concentrated on baby motion analysis from input video data and they collected only papers dealing with general movement assessment (GMA) issues. This paper addresses the more general problem of motion assessment for early diagnosis of neurodevelopmental disorders (NDD) in the first 3 years of life.

From the methodological scouting, it emerged that the approaches relying on handcrafted features and shallow classifiers were mainly exploited for fast recognition of key points (e.g., random ferns [117]) to find the positions of body joints or to analyse movement, e.g., by optical flow. Alternatively, the same task was sometimes achieved by OpenPose framework. Other well-established deep learning strategies were used in a preliminary step as well (e.g., for detecting body parts using U-Net [118]). Finally, deep learning was also exploited for the final classification outcomes (general or clinical-specific) through properly introduced architectures. In particular, the modelling of temporal dependencies is the main task assigned to deep architecture such as LSTM.

Besides, a glimpse of recent advancements in computer vision and machine learning has been provided in order to pave the way towards more effective solutions for the addressed issue. In particular, it emerged that improving deep architectures for motion feature extraction (i.e., by an additional MotionSqueeze module) could be an effective pathway to explore for improving computer-aided diagnosis. Human pose estimation has a great potential to be improved as well, for example, by multi-branch structures for a separate regression of key points.

## Figures and Tables

**Table 1:** Main properties of publicly available datasets. N stands for Newborns, I for Infants and T for Toddlers, Misc for Miscellaneous, NA for Not Applicable, U for Unknown.

Database	Contents	Frame Size	Age Range	Info	Frames	Labels
BabyPose [37]	16 Videos	640 × 480	N	Depth 8 bit/16 bit	16,000	12 Body Landmarks
MINI-RGBD [38]	12 Videos	640 × 480	I	RGB/D	12,000	25 Body Landmarks
SyRIP [41]	Images	Misc	I	RGB	2000	17 Body Landmarks
Dataset [42]	85 Youtube Video URLs	Misc	I	RGB	NA	18 Body Landmarks
SSBD [44]	75 Youtube Video URLs	Misc	NA	RGB	U	Behaviors
MMDB [45]	160 Videos	Misc	T	Multimodal	U	ASD Diagnosis
Tariq [46]	162 Videos	Misc	T	RGB	U	Behaviors
DREAM [47]	3121 Videos	NA	T	Depth	NA	3D Skeleton Gaze ADOS scores
3d-AD [48]	100 Videos	512 × 424	T	Depth	U	Behaviors

**Table 2:** Summarization of works dealing with movements assessment in newborns.

Work	Setup	Input	CV/Ml Task	Clinical Scope
[53,54]	Hospital	Depth	Pose estimation by Keypoints recognition	General
[59]	NICU	Depth	Limb Pose by 2 CNN 2CNN (detection + regression)	General
[55]	Hospital	RGB	Deformable part models	General
[57]	Hospital	Multimodal	Optical Flow + audio features Logistic regression	Normal/Abnormal
[58]	Hospital	RGB	Limb Motion Description by SVM, RF, LDA	WM vs. PR
[60]	NA	Synthetic	Histograms + CNN	Normal/Abnormal

**Table 3:** Summarization of works dealing with movements assessment in Infants.

Work	Setup	Input	Method	Classification Goal
[62]	Home/Hospital	RGB	Motion Feature + Gaussian mixture network	4 type of mov. WMs/FMs/PR/CS
[64]	Hospital	RGB	Motion + MEMD + HT + Decision Tree	CP risk
[63]	Hospital	RGB	OpenPose+NN	FMs
[72]	Treatment Center	RGB	Amount of Motion	Pain Level
[66]	Home/Hospital	RGB	VGG9+LSTM	FMs
[70]	Home/Hospital	RGB	OpenPose+LSTM	FMs
[69]	Home	Smartphone	CIMA-Pose	CP risk

**Table 4:** Summarization of works dealing with movements assessment in Toddlers.

Work	Setup	Input	Method	Goal
[74]	domestic (Tariq dataset)	RGB	Random Forests	Typical/Atypical
[73]	Rehabilitation Environment	Multiview RGB	Faster R-CNN + LSTM + learnable fusion coefficients	4 daily actions
[76]	Domestic	RGB	2D Mask R-CNN + particle filter +CNN classifier	Atypical/Typical Trajectories
[78]	Domestic	RGB (from YouTube)	YOLOv3 + HOF + K-means K-means + MLP	4 repetitive Actions
[77]	Domestic (SSBD dataset)	RGB	CNN + LSTM	ASD/Typical
[79]	domestic (Tariq dataset)	RGB	Various regressors Classifiers	ASD Features Rating

**Table 5:** Recent works on human motion analysis.

Work	Improved Task	Key Contribution
[81]	Motion Features Extraction	Spatiotemporal self-similarity
[82]	Motion Features Extraction	MotionSqueeze module
[83]	Pose Estimation (Key points Positioning)	Multi-branch regression
[84]	Pose Estimation (Key points Positioning)	Cascade Transformers
[85]	Pose Estimation (Key points Positioning)	Adversarial algorithms
[86]	Human Completion	Topological Structure/Memory Bank
[87]	Skeleton-Based Action Recognition	Memory Attention Networks
[90]	Action Recognition	Temporal-Spatial pooling block
[91]	Action Recognition	CNN+Autoencoder+LSTM
[101]	Action Recognition	Contrastive Learning
[100]	Action Recognition	Semi-supervised Action Detection
[95,96,97,98,99]	Action Classification	Transformers
[103]	Action Quality Assessment	Contrastive Regression
[104]	video representation	Space-Time Graph
[105,106]	Video Representation	Self-supervised learning
[107]	Temporal Modeling	Two-level Motion Modeling
[108]	Motion Segment Extraction	Hierarchical Framework
[109]	Temporal Action Localization	E2E anchor free method
[110]	Temporal Action Localization	Anchor-Constrained Viterbi
[111]	Temporal Action Localization	Memory Network
[112]	Temporal Action Localization	Multi-Label Action Dependency layer
[113]	Human Object Interaction	Transformer /Cascade detector
[114]	Human Object Interaction	Graph Networks
